# Spousal Concordance in the Development of Functional Limitations Among Married Adults in China

**DOI:** 10.1001/jamanetworkopen.2021.25577

**Published:** 2021-09-28

**Authors:** Jingwen Wang, Qian Wang, Xiang-Yu Hou, Sunan Chen, Zhen Guo, Wei Du, Lijun Fan

**Affiliations:** 1 Department of Medical Insurance, School of Public Health, Southeast University, Nanjing, China; 2 Guangdong Provincial Geriatrics Institute, Guangdong Provincial People’s Hospital, Guangdong Academy of Medical Sciences, Guangzhou, China; 3 School of Health and Wellbeing, University of Southern Queensland, Ipswich, Queensland, Australia; 4 Department of Epidemiology and Biostatistics, School of Public Health, Southeast University, Nanjing, China

## Abstract

**Importance:**

Functional limitation is increasingly common as people age and is often associated with negative consequences. Evidence of the dynamics of functional limitation within couples in China is still inadequate.

**Objectives:**

To examine whether functional limitation was associated within middle-aged and older couples and to explore sex differences in spousal associations.

**Design, Setting, and Participants:**

In this nationwide, population-based cohort study performed from January 1, 2011, to December 31, 2018, participants were selected using multistage probability sampling, and 5207 community-dwelling couples (10 414 individuals) 45 years or older were included in the nationally representative China Health and Retirement Longitudinal Study. Data analysis was performed from January 1 to February 28, 2021.

**Exposures:**

The exposure variable was the presence of functional limitation in spouses. Functional limitation was measured by the activities of daily living (ADLs) and instrumental activities‎ of daily ‎living (IADLs) scales and was defined as having difficulty in independently performing at least 1 ADL or IADL item.

**Main Outcomes and Measures:**

The main outcome was functional limitation in index participants. Multivariable logistic regression with generalized estimating equations was used to estimate the reciprocal association of functional limitation within couples over time.

**Results:**

A total of 5207 married, different-sex couples (mean [SD] age, 59.1 [8.8] years for husbands and 57.0 [8.2] years for wives) were included in the study. For husbands, the number (percentage) of participants classified with baseline functional limitation was 1140 (21.9%), the number (percentage) with ADL limitation was 684 (13.1%), and the number (percentage) with IADL limitation was 834 (16.0%). For wives, the number (percentage) of participants classified with baseline functional limitation was 1502 (28.8%), the number (percentage) with ADL limitation was 887 (17.0%), and the number (percentage) with IADL limitation was 1183 (22.7%). Longitudinal results demonstrated an association in spouses developing functional limitation (adjusted odds ratio [OR], 2.55; 95% CI, 2.41-2.69; *P* < .001), ADL limitation (adjusted OR, 2.26; 95% CI, 2.11-2.41; *P* < .001), and IADL limitation (adjusted OR, 2.58; 95% CI, 2.43-2.73; *P* < .001). Subgroup analyses by sex revealed similar patterns of spousal health concordance in terms of all studied outcomes, indicating no sex specificity.

**Conclusions and Relevance:**

This population-based cohort study suggests that among Chinese middle-aged and older couples there is significant concordance in the development of functional limitation. This study of spousal functional ability from a dyadic perspective may help in the understanding of health risks within a wider familial context and offers novel insights for prioritizing policy focus from individual centered to couple based.

## Key Points

**Question:**

Are spouses concordant in the development of functional limitation over time in middle and old age?

**Findings:**

In this cohort study of 10 414 community-dwelling participants (5207 married, different-sex couples) 45 years or older in China, significant interdependent associations were observed within a couple in the development of major public health problems, including functional limitation, activities of daily living limitation, and instrumental activities of daily living limitation.

**Meaning:**

In an unprecedentedly aging population accompanied by increasing burden from functional impairment, recognizing the spousal role in shaping health and prioritizing couple-oriented rather than individual-alone public health strategies is warranted for effective prevention and treatment of functional limitations.

## Teaser

This cohort study of middle-aged and older Chinese couples examines whether spouses are concordant in the development of functional limitation over time and whether sex differences exist in spousal health associations.

## Introduction

The World Health Organization reports that developing and maintaining functional ability that enables an individual’s dignity and well-being in older age represents a top priority for healthy aging.^1^ However, functional limitation, a substantial impairment in a person’s ability to effectively perform main daily tasks (such as mobility and personal hygiene),^2,3,4^ is still an increasingly common experience in later life and becomes a significant public health concern worldwide. Extensive studies have documented negative consequences associated with functional limitation, such as depression,^5^ cognitive impairment,^6^ reduced quality of life,^7^ increased health care use and cost,^8,9^ and morbidity and mortality,^10,11,12^ which can impose a heavy burden on families and society. Nevertheless, functional limitation is amenable to interventions,^13^ and therefore a better understanding of its underlying risk factors is critical to develop appropriate countermeasures for mitigating functional loss and its associated poor outcomes.

Although the origin of functional limitation remains unclear, empirical studies^14,15,16,17^ have identified numerous influencing factors, including sociodemographic characteristics, physical and biological status, and lifestyle. The association of one’s own characteristics with functional health is increasingly apparent; however, inadequate data are available on the impact from spouses. Previous literature^18,19^ has suggested that the social context in which the individuals live, including especially their spouses, has the potential to shape a person’s well-being. Spouses are in an intimate relationship and are often the primary caregiver for each other.^20^ They live in a shared environment, gain almost equal access to resources, have similar health behaviors, demonstrate convergent mood, and are exposed to common stressors.^19,21,22,23^ Therefore, spousal health is not supposed to develop in isolation: characteristics of one are likely to influence the other, and spouses form a reasonable and important dyad for evaluating interdependency.

An increasing body of studies have explored the spousal dynamics and reciprocal associations in health or health behaviors among couples, and in general, these studies point to spousal concordance or similarities across a variety of health-related measures, primarily including blood pressure and other biomarkers,^19,24,25,26^ health behaviors,^27,28^ depression and cognitive function,^21,29,30^ chronic illnesses,^31,32,33,34,35,36,37^ and subjective well-being.^38,39^ However, the range of investigated health conditions is still narrow, and relatively little is known about functional limitation. A limited existing evidence examining spousal reciprocal influence on functional health or the broader syndrome of frailty that often contains functional impairment came from the US^34,37,40^ and Korea^41^ but not from China. The available Chinese studies that involve spousal functional health examine only its association with depression^42^ or self-rated health.^43^ Moreover, investigation into sex differences in spousal health concordance has received emerging scholarly attention, but the conclusions remain scarce and contradictory. Some studies^26,33,41,44^ have found sex specificity but were inconclusive toward whether husbands or wives were more sensitive to spousal influence, whereas other studies^45,46^ found no sex differences. Independency or interdependency between spousal health can be largely influenced by both cultural background and gendered roles across different countries,^47^ and more evidence is warranted from China, one of the world’s most populous countries with distinctive socioeconomic and family structure. Therefore, the current study aims to examine whether there is spousal concordance in the development of functional limitation among middle-aged and older couples in China, and further explores sex differences in spousal associations.

## Methods

### Data and Study Sample

**Figure 1.**
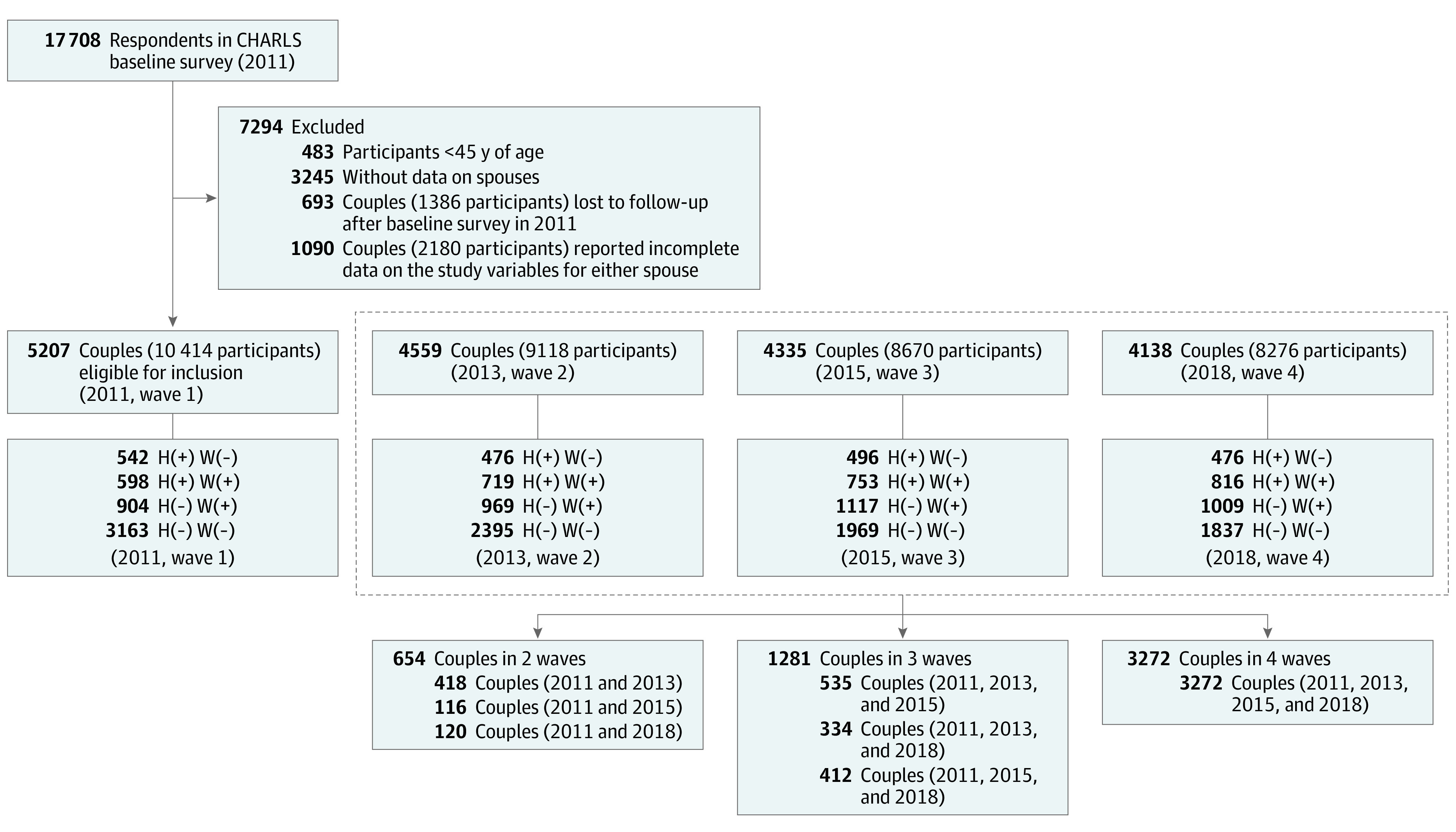
Flowchart of Study Sample From the China Health and Retirement Longitudinal Study (CHARLS) H(+) indicates husbands with functional limitations; H(−), husbands without functional limitations; W(+), wives with functional limitations; W(−), wives without functional limitations.

This cohort study analyzed 4 waves of data (2011, 2013, 2015, and 2018) from the China Health and Retirement Longitudinal Study (CHARLS). CHARLS is a nationally representative survey among approximately 17 000 community-dwelling individuals 45 years or older and their spouses from 28 provinces in China, based on multistage probability sampling and face-to-face interviews via structured questionnaire. Details for CHARLS have been published elsewhere.^48^ Given the study objectives, we chose samples from CHARLS that met the following criteria: (1) individuals were 45 years or older at baseline, (2) both spouses were included, and (3) both spouses had complete records of study variables at baseline and in at least 1 follow-up wave, which finally led to an analytic sample of 5207 couples (10 414 individuals). For each participant, study variables were repeatedly measured at every available time point from January 1, 2011, to December 31, 2018. Data analysis for the current study was performed from January 1 to February 28, 2021. Figure 1 illustrates the sample flowchart. Baseline characteristics were similar between participants with complete data and those with missing data (eTable 1 in the Supplement). The CHARLS survey was conducted in line with the Declaration of Helsinki^49^ and ethically approved by the institutional review board at Peking University. All participants provided written informed consent. All data were deidentified. This study followed the Strengthening the Reporting of Observational Studies in Epidemiology (STROBE) reporting guideline.^50^

### Measurements

#### Functional Limitation

Functional limitation was measured by previously validated scales, including activities of daily living (ADLs) and instrumental activities‎ of daily ‎living (IADLs).^34,51,52^ Participants were asked whether they had difficulties in independently performing 6 ADL activities (namely, dressing, bathing, continence, eating, getting into or out of bed, and toileting) and 5 IADL activities (namely, shopping, doing housework, cooking, taking medications, and managing finances). Answer options included (1) have no difficulty, (2) have some difficulty but can still do it, (3) have difficulty and need help, and (4) cannot do it, which were coded with scores of 0 to 3, respectively. In accordance with previous literature, binary variables of ADL and IADL limitation were constructed, where limitation in ADLs and IADLs was defined if the participant had difficulty in at least 1 of the previously described ADL and IADL activities.^34,51^ The overall functional limitation was further defined if the participant was functionally impaired in either ADL or IADL indicators. Meanwhile, we considered continuous scores of functional limitation (scores ranging from 0 to 33, with higher scores indicating poorer function), ADL limitation (scores ranging from 0 to 18, with higher scores indicating poorer function), and IADL limitation (scores ranging from 0 to 15 scores, with higher scores indicating poorer function) by summing the score of each response to items that constructed the 3 scales.

#### Covariates

The following covariates were considered: age, residence (rural and urban), region of location (Eastern, Central, and Western China), occupation (agricultural and nonagricultural work), educational level (illiterate, literate but did not finish primary school, primary school, middle school, and high school and above), household income per capita (four quartiles), health insurance (no insurance and different types of insurance), social activities (no and yes), smoking (never, current smoker, and former smoker), drinking (never, drink but not more than once per month, and drink more than once per month), self-rated health (good, fair, and poor) and multimorbidity (the presence of 0, 1, and ≥2 chronic diseases).

### Statistical Analysis

Stata software, version 16.0 (StataCorp LLC) was used to manage and analyze data. Baseline characteristics are presented as numbers (percentages) for categorical variables. We performed the McNemar χ^2^ test to examine the differences within couples in the sociodemographic characteristics and the χ^2^ test of independent groups to test the differences in functional outcomes across various characteristic groups.

Logistic regression with the generalized estimating equation (GEE) method was used to estimate the reciprocal associations in functional limitation, ADL limitation, or IADL limitation within couples over time, where the results are presented as odds ratios (ORs) with 95% CIs. The GEE method was used because it is suitable for analyzing repeated measures in longitudinal studies and is commonly used in situations in which the normal assumption of independent observations is not met.^53,54,55^ The GEE method allows us to obtain robust risk estimates that account for the within-participant association across repeated measures or clustering at households, and it also fits when the repeated observations are not at equally spaced or the same intervals for all participants.^55,56^ The Stata xtgee module was applied to fit the models, with working association structure specified as exchangeable. Four GEE models were hierarchically established to illustrate possible confounding: model 1 was unadjusted; model 2 was adjusted for age, residence, region, occupation, educational level, income, and health insurance; model 3 additionally adjusted for behavioral covariates, including social activities, smoking, and drinking; and model 4 additionally adjusted for self-rated health and multimorbidity.

Stratified analyses according to sex in total sample and in different age groups (middle-aged couples and elderly couples) were further performed using the GEE models. We assessed sex differences by interaction tests. We also conducted sensitivity analysis by treating functional limitations as continuous scores, using GEE linear regression models to assess associations and interaction tests to explore sex differences. A 2-sided *P* < .05 was considered statistically significant.

## Results

### Baseline Sample Characteristics

A total of 5207 married, different-sex couples (mean [SD] age, 59.1 [8.8] years for husbands and 57.0 [8.2] years for wives) were included in the study. There were 64 812 person-years of follow-up (mean of 6.22 person-years per participant), with a median follow-up period of 7 years (interquartile range, 4-7 years). For husbands, the number (percentage) of participants classified with baseline functional limitation was 1140 (21.9%), the number (percentage) with ADL limitation was 684 (13.1%), and the number (percentage) with IADL limitation was 834 (16.0%). For wives, the number (percentage) of participants classified with baseline functional limitation was 1502 (28.8%), the number (percentage) with ADL limitation was 887 (17.0%), and the number (percentage) with IADL limitation was 1183 (22.7%). Baseline characteristics are listed in Table 1. Results from the McNemar χ^2^ test indicated that husbands were relatively older (age ≥75 years: 290 [5.57%] men vs 157 [3.02%] women; *P* < .001), better educated (high school and above: 878 [16.86%] men vs 454 [8.72%] women; *P* < .001), and more likely to have urban residence (1188 [22.82%] women vs 919 [17.65%]; *P* < .001), take on agricultural work (3185 [61.17%] vs 3008 [57.77%]; *P* < .001), participate in social activities (2662 [51.12%] vs 2541 [48.80%]; *P* = .003), smoke (current smokers: 3000 [57.61%] vs 297 [5.70%]; *P* < .001), drink alcohol (more than once a month: 2406 [46.21%] vs 358 [6.88%]; *P* < .001), have good self-rated health (1374 [26.39%] vs 1054 [20.24%]; *P* < .001), and be absent of comorbidity (1821 [34.97%] vs 1671 [32.09%]; *P* < .001) than their wives. Results from the χ^2^ test of independent groups indicated that both husbands and wives with functional limitation were older (55-65 years of age: 476 [22.18%] men and 672 [31.59%] women; *P* < .001), more often had a rural residence (942 [23.44%] men and 1335 [31.13%] women; *P* < .001), more often lived in non-Eastern China (349 [22.65%] men in Central China and 394 [23.79%] men in Western China; *P* = .009; 464 [30.11%] women in Central China and 516 [31.16%] in Western China; *P* < .001), were more poorly educated (illiterate: 215 [35.42%] men vs 764 [38.03%] women; *P* < .001), were more economically disadvantaged (poorest household income: 395 [30.15%] men vs 475 [36.26%] women; *P* < .001), were less engaged in social activities (652 [25.62%] men vs 886 [33.23%]; *P* < .001), were former smokers (240 [28.78%] men vs 42 [49.41%] women; *P* < .001), and reported poor health (564 [43.89%] vs 848 [49.45%]; *P* < .001) and having 2 or more chronic diseases (584 [34.05%] vs 794 [41.33%]; *P* < .001) than those without impairments.

**Table 1.** Baseline Characteristics of Study Participants According to Functional Limitation Status

Characteristic	No. (%) of study participants								*P* value (for pairs)^a^
	Husband				Wife				
	Overall (n = 5207)	Functional limitation (n = 1140)	ADL limitation (n = 684)	IADL limitation (n = 834)	Overall (n = 5207)	Functional limitation (n = 1502)	ADL limitation (n = 887)	IADL limitation (n = 1183)	
Age, y									
45-55	1746 (33.53)	219 (12.54)	111 (6.36)	162 (9.28)	2153 (41.35)	425 (19.74)	235 (10.92)	330 (15.33)	<.001
55-65	2146 (41.21)	476 (22.18)	276 (12.86)	344 (16.03)	2127 (40.85)	672 (31.59)	388 (18.24)	533 (25.06)	
65-75	1025 (19.69)	329 (32.10)	219 (21.37)	239 (23.32)	770 (14.79)	316 (41.04)	206 (26.75)	244 (31.69)	
≥75	290 (5.57)	116 (40.00)	78 (26.90)	89 (30.69)	157 (3.02)	89 (56.69)	58 (36.94)	76 (48.41)	
*P* value^b^	NA	<.001	<.001	<.001	NA	<.001	<.001	<.001	
Residence									
Rural	4019 (77.18)	942 (23.44)	559 (13.91)	695 (17.29)	4288 (82.35)	1335 (31.13)	788 (18.38)	1054 (24.58)	<.001
Urban	1188 (22.82)	198 (16.67)	125 (10.52)	139 (11.70)	919 (17.65)	167 (18.17)	99 (10.77)	129 (14.04)	
*P* value^b^	NA	<.001	.002	<.001	NA	<.001	<.001	<.001	
Region									
Eastern China	2010 (38.60)	397 (19.75)	211 (10.50)	296 (14.73)	2010 (38.60)	522 (25.97)	300 (14.93)	409 (20.35)	NA
Central China	1541 (29.59)	349 (22.65)	235 (15.25)	236 (15.31)	1541 (29.59)	464 (30.11)	278 (18.04)	360 (23.36)	
Western China	1656 (31.80)	394 (23.79)	238 (14.37)	302 (18.24)	1656 (31.80)	516 (31.16)	309 (18.66)	414 (25.00)	
*P* value^b^	NA	.009	<.001	.01	NA	.001	.005	.003	
Occupation									
Agricultural work	3185 (61.17)	673 (21.13)	381 (11.96)	473 (14.85)	3008 (57.77)	865 (28.76)	476 (15.82)	666 (22.14)	<.001
Nonagricultural work	2022 (38.83)	467 (23.10)	303 (14.99)	361 (17.85)	2199 (42.23)	637 (28.97)	411 (18.69)	517 (23.51)	
*P* value^b^	NA	.10	.002	.004	NA	.87	.007	.24	
Educational level									
Illiterate	607 (11.66)	215 (35.42)	137 (22.57)	182 (29.98)	2009 (38.58)	764 (38.03)	441 (21.95)	634 (31.56)	<.001
Literate	926 (17.78)	286 (30.89)	166 (17.93)	209 (22.57)	954 (18.32)	327 (34.28)	204 (21.38)	247 (25.89)	
Primary school	1390 (26.69)	310 (22.30)	201 (14.46)	207 (14.89)	925 (17.76)	220 (23.78)	126 (13.62)	165 (17.84)	
Middle school	1406 (27.00)	236 (16.79)	131 (9.32)	171 (12.16)	865 (16.61)	144 (16.65)	82 (9.48)	109 (12.60)	
High school and above	878 (16.86)	93 (10.59)	49 (5.58)	65 (7.40)	454 (8.72)	47 (10.35)	34 (7.49)	28 (6.17)	
*P* value^b^	NA	<.001	<.001	<.001	NA	<.001	<.001	<.001	
Household income									
Quartile 1 (poorest)	1310 (25.16)	395 (30.15)	233 (17.79)	298 (22.75)	1310 (25.16)	475 (36.26)	300 (22.90)	381 (29.08)	NA
Quartile 2	1291 (24.79)	313 (24.24)	197 (15.26)	233 (18.05)	1291 (24.79)	413 (31.99)	241 (18.67)	328 (25.41)	
Quartile 3	1283 (24.64)	246 (19.17)	147 (11.46)	174 (13.56)	1283 (24.64)	373 (29.07)	210 (16.37)	284 (22.14)	
Quartile 4 (richest)	1323 (25.41)	186 (14.06)	107 (8.09)	129 (9.75)	1323 (25.41)	241 (18.22)	136 (10.28)	190 (14.36)	
*P* value^b^	NA	<.001	<.001	<.001	NA	<.001	<.001	<.001	
Health insurance									
No	264 (5.07)	54 (20.45)	36 (13.64)	35 (13.26)	285 (5.47)	81 (28.42)	48 (16.84)	61 (21.4)	<.001
NRCMS	3850 (73.94)	909 (23.61)	541 (14.05)	667 (17.32)	4114 (79.01)	1282 (31.16)	759 (18.45)	1013 (24.62)	
UEBMI	637 (12.23)	96 (15.07)	59 (9.26)	69 (10.83)	419 (8.05)	47 (11.22)	28 (6.68)	34 (8.11)	
URBMI	249 (4.78)	50 (20.08)	29 (11.65)	40 (16.06)	282 (5.42)	71 (25.18)	38 (13.48)	59 (20.92)	
Others	207 (3.98)	31 (14.98)	19 (9.18)	23 (11.11)	107 (2.05)	21 (19.63)	14 (13.08)	16 (14.95)	
*P* value^b^	NA	<.001	.006	<.001	NA	<.001	<.001	<.001	
Social activities									
No	2545 (48.88)	652 (25.62)	410 (16.11)	494 (19.41)	2666 (51.20)	886 (33.23)	519 (19.47)	718 (26.93)	.003
Yes	2662 (51.12)	488 (18.33)	274 (10.29)	340 (12.77)	2541 (48.80)	616 (24.24)	368 (14.48)	465 (18.30)	
*P* value^b^	NA	<.001	<.001	<.001	NA	<.001	<.001	<.001	
Smoking									
Never	1373 (26.37)	293 (21.34)	177 (12.89)	223 (16.24)	4825 (92.66)	1349 (27.96)	800 (16.58)	1054 (21.84)	<.001
Current	3000 (57.61)	607 (20.23)	350 (11.67)	431 (14.37)	297 (5.70)	111 (37.37)	58 (19.53)	94 (31.65)	
Former	834 (16.02)	240 (28.78)	157 (18.82)	180 (21.58)	85 (1.63)	42 (49.41)	29 (34.12)	35 (41.18)	
*P* value^b^	NA	<.001	<.001	<.001	NA	<.001	<.001	<.001	
Drinking									
None	2240 (43.02)	563 (25.13)	361 (16.12)	419 (18.71)	4603 (88.40)	1322 (28.72)	785 (17.05)	1039 (22.57)	<.001
Once/mo or less	561 (10.77)	97 (17.29)	57 (10.16)	65 (11.59)	246 (4.72)	62 (25.20)	32 (13.01)	49 (19.92)	
More than once/mo	2406 (46.21)	480 (19.95)	266 (11.06)	350 (14.55)	358 (6.88)	118 (32.96)	70 (19.55)	95 (26.54)	
*P* value^b^	NA	<.001	<.001	<.001	NA	.10	.11	.13	
Self-rated health									
Good	1374 (26.39)	123 (8.95)	52 (3.78)	89 (6.48)	1054 (20.24)	125 (11.86)	49 (4.65)	107 (10.15)	<.001
Fair	2548 (48.93)	453 (17.78)	248 (9.73)	313 (12.28)	2438 (46.82)	529 (21.70)	267 (10.95)	397 (16.28)	
Poor	1285 (24.68)	564 (43.89)	384 (29.88)	432 (33.62)	1715 (32.94)	848 (49.45)	571 (33.29)	679 (39.59)	
*P* value^b^	NA	<.001	<.001	<.001	NA	<.001	<.001	<.001	
Multimorbidity									
0	1821 (34.97)	231 (12.69)	101 (5.55)	185 (10.16)	1671 (32.09)	282 (16.88)	121 (7.24)	218 (13.05)	<.001
1	1671 (32.09)	325 (19.45)	197 (11.79)	225 (13.46)	1615 (31.02)	426 (26.38)	242 (14.98)	332 (20.56)	
≥2	1715 (32.94)	584 (34.05)	386 (22.51)	424 (24.72)	1921 (36.89)	794 (41.33)	524 (27.28)	633 (32.95)	
*P* value^b^	NA	<.001	<.001	<.001	NA	<.001	<.001	<.001	

Abbreviations: ADLs, activities of daily living; IADLs, instrumental activities‎ of daily ‎living; NA, not applicable; NRCMS, New Rural Cooperative Medical Scheme; UEBMI, Urban Employee Basic Medical Insurance; URBMI, Urban Resident Basic Medical Insurance.

^a^ McNemar χ^2^ test was used to examine the differences within couples in the characteristics.

^b^ χ^2^ Test of independent groups was used to examine differences in outcomes across characteristic groups.

### Spousal Concordance in Functional Limitation Over Time

Table 2 presents the longitudinal results on spousal associations in functional limitation. Significant concordance was prospectively demonstrated within couple pairs in functional limitation (adjusted OR, 2.55; 95% CI, 2.41-2.69), ADL limitation (OR, 2.26; 95% CI, 2.11-2.41), and IADL limitation (OR, 2.58; 95% CI, 2.43-2.73), after full adjustment for covariates, including age, residence, region, occupation, educational level, income, insurance, social activities, smoking, drinking, self-rated health, and multimorbidity. This remained the case in the crude model without any adjustment and in the partially adjusted models.

**Table 2.** Reciprocal Association in Functional Limitation Among 5207 Middle-aged and Older Couples, 2011-2018

Outcomes	Model adjusting for sex, total^a^		Sex interaction models				
			Husband → wife		Wife → husband		*P* value for sex interaction^b^
	OR (95% CI)	*P* value	OR (95% CI)	*P* value	OR (95% CI)	*P* value	
Functional limitation							
Model 1^c^	2.93 (2.79-3.08)	<.001	2.99 (2.78-3.21)	<.001	2.88 (2.68-3.08)	<.001	.59
Model 2^d^	2.56 (2.43-2.70)	<.001	2.61 (2.42-2.81)	<.001	2.55 (2.36-2.74)	<.001	.54
Model 3^e^	2.56 (2.43-2.70)	<.001	2.60 (2.41-2.80)	<.001	2.56 (2.37-2.76)	<.001	.61
Model 4^f^	2.55 (2.41-2.69)	<.001	2.58 (2.38-2.79)	<.001	2.55 (2.36-2.76)	<.001	.57
ADL limitation							
Model 1^c^	2.58 (2.42-2.75)	<.001	2.63 (2.40-2.88)	<.001	2.53 (2.32-2.76)	<.001	.69
Model 2^d^	2.30 (2.15-2.45)	<.001	2.33 (2.12-2.56)	<.001	2.28 (2.08-2.49)	<.001	.96
Model 3^e^	2.30 (2.15-2.45)	<.001	2.32 (2.11-2.55)	<.001	2.29 (2.09-2.51)	<.001	.95
Model 4^f^	2.26 (2.11-2.41)	<.001	2.26 (2.05-2.48)	<.001	2.28 (2.07-2.50)	<.001	.74
IADL limitation							
Model 1^c^	3.02 (2.86-3.19)	<.001	3.07 (2.84-3.31)	<.001	2.98 (2.76-3.21)	<.001	.67
Model 2^d^	2.59 (2.45-2.75)	<.001	2.64 (2.43-2.86)	<.001	2.60 (2.39-2.81)	<.001	.63
Model 3^e^	2.59 (2.45-2.75)	<.001	2.62 (2.41-2.85)	<.001	2.61 (2.41-2.83)	<.001	.70
Model 4^f^	2.58 (2.43-2.73)	<.001	2.61 (2.39-2.84)	<.001	2.60 (2.39-2.83)	<.001	.64

Abbreviations: ADLs, activities of daily living; IADLs, instrumental activities‎ of daily ‎living; OR, odds ratio.

^a^ In models for the total sample, sex was additionally added to the models as an adjustment variable (models 1-4).

^b^ *P* for sex interaction was examined using the sex × functional limitation (or ADL and IADL limitation) interaction test.

^c^ Model 1 was not adjusted for any covariates.

^d^ Model 2 was adjusted for individual’s age, residence, region, occupation, educational level, household income, and health insurance.

^e^ Model 3 was adjusted for individual’s age, residence, region, occupation, educational level, household income, health insurance, social activities, smoking, and drinking.

^f^ Model 4 was adjusted for individual’s age, residence, region, occupation, educational level, household income, health insurance, social activities, smoking, drinking, self-rated health, and multimorbidity.

### Stratification Analysis by Sex 

Table 2 also presents results on subgroup analyses by sex. After fully adjusting for the predefined covariates, the husband’s functional limitation was significantly associated with the wife’s functional limitation (OR, 2.58; 95% CI, 2.38-2.79), and the wife’s functional limitation was also significantly associated with the husband’s functional limitation (OR, 2.55; 95% CI, 2.36-2.76), indicating a similar spousal concordance among women and men (*P* = .57 for interaction). Consistent patterns were observed for the other 2 outcomes, indicating that spousal concordance in ADL or IADL limitation similarly existed irrespective of sex (ADL limitation, husbands to wives: OR, 2.26; 95% CI, 2.05-2.48, wives to husbands: OR, 2.28; 95% CI, 2.07-2.50; IADL limitation, husbands to wives: OR, 2.61; 95% CI, 2.39-2.84, wives to husbands: OR, 2.60; 95% CI, 2.39-2.83).

**Figure 2.**
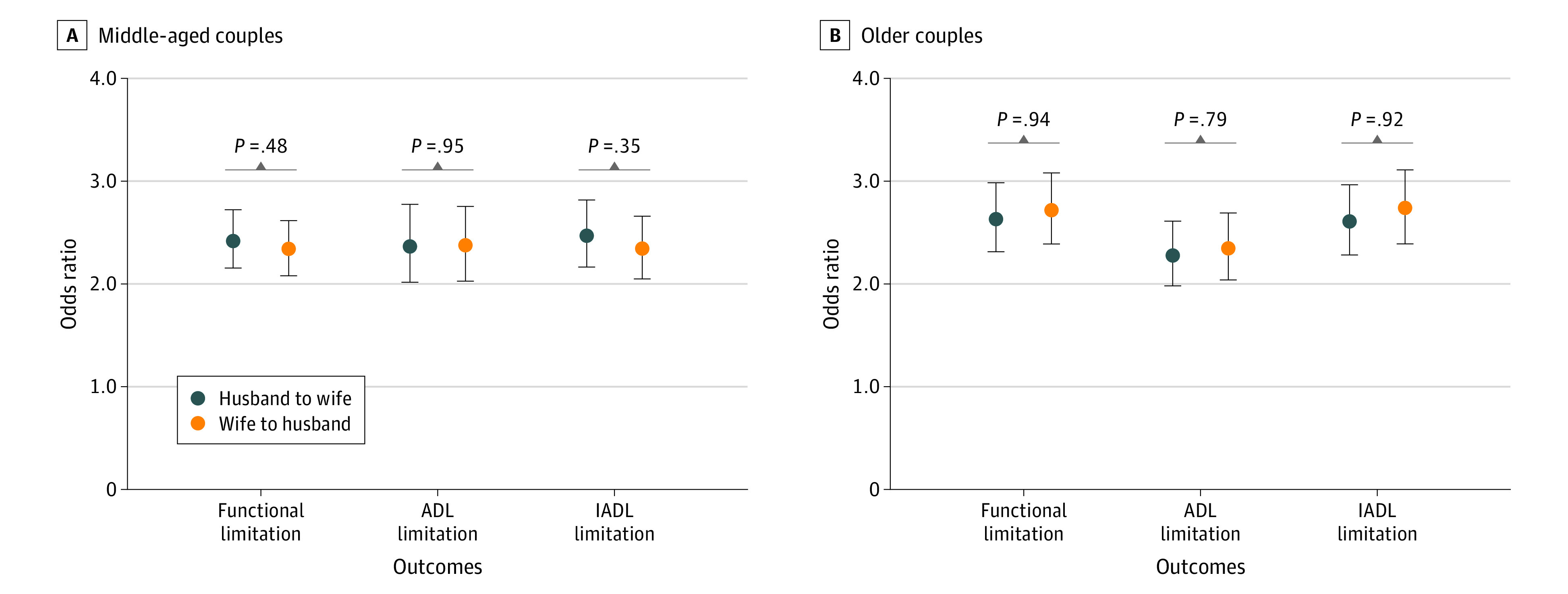
Reciprocal Association in Functional Limitation by Sex Among Different Age Groups, 2011-2018 All models were adjusted for individual’s residence, region, occupation, educational level, household income, health insurance, social activities, smoking, drinking, self-rated health, and multimorbidity. Middle-aged couples were 45 to 60 years of age; older couples were 60 years of age or older. The sex interaction term *P* was obtained using the sex × functional limitation (or activities of daily living [ADL] or instrumental activities‎ of daily ‎living [IADL] limitation) interaction test. Error bars indicate 95% CIs.

We further investigated sex differences in spousal health associations in 2 age groups (Figure 2). Among both middle-aged couples (45-59 years of age) and elderly couples (≥60 years of age), the husband’s functional limitation was significantly associated with the wife’s functional limitation over time and vice versa. The extent of the negative association with functional limitation from husbands to wives appeared similar as did the reverse (middle age: OR, 2.42 [95% CI, 2.15-2.72] vs 2.33 [95% CI, 2.08-2.61]; *P* = .48 for interaction; old age: OR, 2.62 [95% CI, 2.31-2.98] vs 2.71 [95% CI, 2.39-3.08]; *P* = .94 for interaction), indicating no sex specificity of spousal health concordance in both middle and old age. Such findings from stratification analyses remained consistent when we examined 2 other outcomes of ADL and IADL limitation.

### Sensitivity Analysis

Results from analyses treating functional limitations as continuous variables are given in eTable 2 and eFigure in the Supplement. The levels of functional limitations (or ADL and IALD limitations) were significantly associated among couples, and sex did not significantly moderate spousal associations (functional limitation in unadjusted model: husband to wife: β = 0.13; 95% CI, 0.10-0.15; *P* < .001; wife to husband: β = 0.13; 95% CI, 0.11-0.15; *P* < .001; *P* = .73 for sex interaction) (eTable 2 and eFigure in the Supplement).

## Discussion

To our knowledge, this cohort study is the first nationally representative panel data analysis that used a dyadic approach to examine spousal associations of functional limitation in China. We found evidence that suggested health similarities or concordance in the development of functional limitation (or ADL and IADL limitation) within middle-aged and older couples. In addition, the partner association in functional impairment remained evident and similar among women and men.

Our finding of spousal concordance in functional limitation was consistent with previous studies.^21,24,25,26,29,30,31,32,33,34,35,36,37,38,39,40,41^ For example, 2 studies^34,37^ in the US found that 1 spouse’s functional decline was significantly correlated with the other spouse’s functional decline, but the studied participants were limited to couples 70 years or older. Two other relevant studies^40,41^ from the US and Korea on frailty, a geriatric syndrome that often included evaluation of functional ability, demonstrated spousal interdependency in frailty as well. A variety of other studies, although not focusing on functional health, also revealed health similarities in couples with regard to biomarker change,^24,25,26^ mental health,^21,29,30^ cardiovascular diseases,^31,32,35^ sensory impairment,^33,36^ and subjective well-being.^38,39^ Our study adds to the existing literature, given that previous research was sometimes limited because of lack of studies conducted in China, investigation of only patients with a particular disease or residents in small geographic areas, using partner-reported information rather than paired data, small sample size, or cross-sectional design that failed to determine the chronological sequence of events. The findings of spousal health concordance might be explained through multiple theories or mechanisms as follows. First, the assortative mating hypothesis suggests that individuals are instinctively attracted to and will want to marry a spouse with similar characteristics, such as social background, personality, life attitudes, and behaviors.^57^ Second, the shared resource hypothesis proposes that the features of a couple tend to converge over time because of their shared resources to counteract stress, such as living environment, financial resources, and social networks, as well as their shared experiences of stress.^27,58^ Third, the emotional contagion theory suggests that the low mood of an ill partner may spread to spouses who are in close contact, which becomes a risk factor for spousal health.^36,38^ Fourth, the caregiver burden hypothesis indicates that providing support to an ill spouse can be physically and emotionally stressful, which may negatively affect the caregiver’s well-being.^59,60^ Fifth, there is also the possibility that the index individuals become more aware of functional limitations (that might have always been there but were undernoticed) after their spouse officially reports a functional limitation, suggesting that the association could be in part associated with increased reporting instead of true concordance. However, lack of causal factors in the CHARLS data in relation to these hypotheses restricted our ability to explore further.

Both husbands and wives, irrespective of sex, were found to display significant health concordance with their partners in our study. Some previous studies^45,46,61^ concluded similar findings that suggested no sex specificity in spousal interdependency, whereas others^41,44,62,63,64,65,66^ documented discrepant findings that support sex differences, even though they were also inconclusive on which sex was more sensitive to spousal influence. For instance, some research indicated that husbands were more responsive to spousal chronic diseases than wives^44,62,63^; in contrast, some indicated that wives were more susceptible to their husband’s illness, such as frailty, metabolic syndrome, and depression, than vice versa.^41,64,65,66^ We speculate that the following explanations may account for the equivocal results. On the one hand, husbands are likely to have health similarities to those of their wives because husbands often rely on care from their spouses.^63^ If wives fall ill, husbands may not access adequate care, which thus negatively affects their health.^44,63^ On the other hand, there is also the possibility that wives are vulnerable to their husbands’ health because women are usually more sensitive to others’ negative emotions when facing illness stressors and often take responsibility of providing care for their partners, which may in turn aggravate their own health.^67,68^ Discrepancies in sex roles across studies may be a mixed and complex consequence that results from different gendered roles, cultural varieties, and other subtle contextual factors.^47^ Future research is warranted to obtain a more comprehensive disentanglement of the different spousal effects by sex.

The current study contributes to the existing literature by investigating whether functional ability is associated within a couple and if the association is equal for different sexes. Our findings have important clinical and policy implications. Given the general consensus that healthy aging is more than the absence of disease, functional independence indeed serves as a particularly sensitive and vital marker of health for people with advancing age.^1^ In China, we are currently experiencing accelerating population aging accompanied by increasing burden from functional impairment, which often leads to elevated risks for disability, economic burden, and poor quality of life.^7,9,69^ Understanding functional impairment risks, especially in middle age and old age, has thus become indispensable for measuring future health needs and directing appropriate public health investments. We found in this study that the wider context inclusive of spouses is necessary to consider when studying health; however, the available interventions currently are generally aimed at the affected person but pay little attention to family members. This lack of family member consideration amplifies the need to recognize the role of spouses in shaping health and to prioritize couple-based rather than patient-only public health strategies for effective prevention and treatment of functional problems.

### Strengths and Limitations

Major strengths of our study include the prospective dyadic design based on a large-scale nationwide sample and the particular focus on concordant outcomes within couples. Several limitations also need to be considered. First, the use of self-reported measures may result in recall bias, although this method has been widely adopted in epidemiologic research.^52,70^ Second, because of data unavailability, we were unable to determine the marital intimacy between couples or whether spouses were the primary caregiver for each other, which might also affect spousal functional limitation. Third, in this study, we were unable to rule out the possibility that the increase in functional limitation may be related to more awareness, which warrants further targeted research. Last, it is likely that the results may be different between couples with different follow-up times, but GEE methods were used to fit the population-averaged models. Interpretation of these results thus requires caution in this regard.

## Conclusions

Community-dwelling middle-aged and older couples in China have significant concordance in the development of functional limitation over time, and such spousal associations is similarly observed among women and men, indicating no sex specificity. The study’s focus on investigating married couples’ functional health from a prospective dyadic perspective allows a more comprehensive understanding into health risks within a wider familial context and is crucial for future enhancement of appropriate support systems that shift from an individual-centered to couple-based emphasis. Public health strategies to promote functional independence may benefit from the innovation of targeting spousal health similarities and developing tailored couple-oriented interventions.

## Notes

Supplement. **eTable 1.** Comparison of Baseline Characteristics Between 5207 Couples With Complete Data and 6297 With Missing Data **eTable 2.** Reciprocal Association in Functional Limitation (as Continuous Scores) Among Middle-aged and Older Couples, 2011-2018 (N = 5207 Couples) **eFigure.** Reciprocal Association in Functional Limitation (as Continuous Scores) by Gender Among Different Age Groups, 2011-2018
