# Supplementary material for: Spousal Concordance in the Development of Functional Limitations Among Married Adults in China
Source: JAMA Netw Open. 2021 Sep 28;4(9):e2125577. doi: 10.1001/jamanetworkopen.2021.25577 (PMC8479583; doi:10.1001/jamanetworkopen.2021.25577)
Supplement: Supplement. — eTable 1. Comparison of Baseline Characteristics Between 5207 Couples With Complete Data and 6297 With Missing Data eTable 2. Reciprocal Association in Functional Limitation (as Continuous Scores) Among Middle-aged and Older Couples, 2011-2018 (N = 5207 Couples) eFigure. Reciprocal Association in Functional Limitation (as Continuous Scores) by Gender Among Different Age Groups, 2011-2018 [file jamanetwopen-e2125577-s001.pdf]

Supplementary Online Content

Wang J, Wang Q, Hou XY, et al. Spousal concordance in the development of functional limitations among married adults in China. *JAMA Netw Open*. 2021;4(9):e2125577. doi:10.1001/jamanetworkopen.2021.25577

eTable 1. Comparison of Baseline Characteristics Between 5207 Couples With Complete Data and 6297 With Missing Data

eTable 2. Reciprocal Association in Functional Limitation (as Continuous Scores) Among Middle-aged and Older Couples, 2011-2018 (N = 5207 Couples)

eFigure. Reciprocal Association in Functional Limitation (as Continuous Scores) by Gender Among Different Age Groups, 2011-2018

This supplementary material has been provided by the authors to give readers additional information about their work.

© 2021 Wang J et al. *JAMA Network Open.*

eTable 1. Comparison of Baseline Characteristics Between 5207 Couples With Complete Data and 6297 With Missing Data

Characteristics	Husband (n, %)				Wife (n, %)		
	Participants with complete data (n=5,207)	Participants with incomplete data (n=6,297)	*P* value[a]		Participants with complete data (n=5,207)	Participants with incomplete data (n=6,297)	*P* value[a]
**Age, year**			.573				.266
45-55	1746 (33.53)	2184 (34.71)			2153 (41.35)	2717 (43.16)	
55-65	2146 (41.21)	2570 (40.84)			2127 (40.85)	2506 (39.81)	
65-75	1025 (19.69)	1203 (19.12)			770 (14.79)	892 (14.17)	
≥75	290 (5.57)	336 (5.34)			157 (3.02)	180 (2.86)	
Missing	0	4			0	2	
**Residence**			.564				.781
Rural	4019 (77.18)	4881 (77.64)			4288 (82.35)	5189 (82.55)	
Urban	1188 (22.82)	1406 (22.36)			919 (17.65)	1097 (17.45)	
Missing	0	10			0	11	
**Region**			.904				.886
Eastern	2010 (38.60)	2455 (39.01)			2010 (38.60)	2457 (39.05)	
Central	1541 (29.59)	1851 (29.41)			1541 (29.59)	1850 (29.40)	
Western	1656 (31.80)	1987 (31.57)			1656 (31.80)	1985 (31.55)	
Missing	0	4			0	5	
**Occupation**			.051				.318
Agricultural	3185 (61.17)	3724 (59.38)			3008 (57.77)	3564 (56.84)	
Non-	2022 (38.83)	2548 (40.63)			2199 (42.23)	2706 (43.16)	
Missing	0	25			0	27	
**Education**			.998				.998
Illiterate	607 (11.66)	734 (11.68)			2009 (38.58)	2411 (38.37)	
Literate	926 (17.78)	1116 (17.76)			954 (18.32)	1158 (18.43)	
Primary	1390 (26.69)	1665 (26.50)			925 (17.76)	1128 (17.95)	
Middle	1406 (27.00)	1713 (27.26)			865 (16.61)	1043 (16.60)	
High and	878 (16.86)	1055 (16.79)			454 (8.72)	544 (8.66)	
Missing	0	14			0	13	
**Household**			.952				.952
Quartile	1310 (25.16)	1572 (25.19)			1310 (25.16)	1572 (25.19)	
Quartile 2	1291 (24.79)	1532 (24.55)			1291 (24.79)	1532 (24.55)	
Quartile 3	1283 (24.64)	1548 (24.80)			1283 (24.64)	1548 (24.80)	
Quartile 4	1323 (25.41)	1589 (25.46)			1323 (25.41)	1589 (25.46)	
Missing	0	56			0	56	
**Insurance**			.917				.817
No	264 (5.07)	339 (5.42)			285 (5.47)	374 (5.98)	
NRCMS	3850 (73.94)	4629 (73.95)			4114 (79.01)	4932 (78.86)	
UEBMI	637 (12.23)	759 (12.12)			419 (8.05)	495 (7.91)	
URBMI	249 (4.78)	296 (4.73)			282 (5.42)	330 (5.28)	
Others	207 (3.98)	237 (3.79)			107 (2.05)	123 (1.97)	
Missing	0	37			0	43	
**Social activities**			.642				.547
No	2545 (48.88)	2762 (48.43)			2666 (51.20)	3020 (50.63)	
Yes	2662 (51.12)	2941 (51.57)			2541 (48.80)	2945 (49.37)	
Missing	0	594			0	332	

© 2021 Wang J et al. *JAMA Network Open*.

**eTable 1** (Continued)							
**Characteristics**	**Husband (n, %)**				**Wife (n, %)**		
	**Participants with complete**	**Participants with incomplete**	***P* value[a]**		**Participants with complete**	**Participants with incomplete**	***P* value[a]**
**Smoking**			.172				.594
Never	1373 (26.37)	1632 (27.96)			4825 (92.66)	5829 (93.14)	
Current	3000 (57.61)	3289 (56.35)			297 (5.70)	331 (5.29)	
Former	834 (16.02)	916 (15.69)			85 (1.63)	98 (1.57)	
Missing	0	460			0	39	
**Drinking**			.818				.917
None	2240 (43.02)	2683 (42.76)			4603 (88.40)	5538 (88.24)	
≤once/month	561 (10.77)	699 (11.14)			246 (4.72)	307 (4.89)	
>once/month	2406 (46.21)	2892 (46.09)			358 (6.88)	431 (6.87)	
Missing	0	23			0	21	
**Self-rated**			.747				.893
Good	1374 (26.39)	1696 (27.00)			1054 (20.24)	1293 (20.59)	
Fair	2548 (48.93)	3039 (48.38)			2438 (46.82)	2934 (46.72)	
Poor	1285 (24.68)	1546 (24.61)			1715 (32.94)	2053 (32.69)	
Missing	0	16			0	17	
**Multimorbidity**			.486				.672
0	1821 (34.97)	2265 (36.05)			1671 (32.09)	2059 (32.82)	
1	1671 (32.09)	1985 (31.59)			1615 (31.02)	1941 (30.94)	
≥2	1715 (32.94)	2033 (32.36)			1921 (36.89)	2274 (36.24)	
Missing	0	14			0	23	
**Functional**			.452				.925
Yes	1,140(21.89)	1,322 (21.31)			1,502(28.85)	1,776 (28.77)	
No	4,067(78.11)	4,881 (78.69)			3,705(71.15)	4,398 (71.23)	
Missing	0	94			0	123	
**ADL limitation**			.644				.695
Yes	684(13.14)	797 (12.84)			887(17.03)	1,037 (16.76)	
No	4,523(86.86)	5,408 (87.16)			4,320(82.97)	5,151 (83.24)	
Missing	0	92			0	109	
**IADL limitation**			.201				.691
Yes	834(16.02)	967 (15.45)			1,183(22.72)	1,398 (22.41)	
No	4,373(83.98)	5,292 (84.55)			4,024(77.28)	4,841 (77.59)	
Missing	0	38			0	58	
[a]: *P* values were obtained by chi-square tests. Abbreviations: UEBMI=Urban Employee Basic Medical Insurance, URBMI=Urban Resident Basic Medical Insurance, NRCMS=New Rural Cooperative Medical Scheme							

© 2021 Wang J et al. *JAMA Network Open.*

eTable 2. Reciprocal Association in Functional Limitation (as Continuous Scores) Among Middle-aged and Older Couples, 2011-2018 (N = 5207 Couples)

Outcomes	Model adjusting for gender[e]			Gender interaction models				
	Total			Husband → Wife		Wife → Husband		*P* for gender interaction[f]
	β (95%CI)	*P* value		β (95%CI)	*P* value	β (95%CI)	*P* value	
**Functional limitation**								
Model 1[a]	0.13 (0.11-0.14)	<.001		0.13 (0.10-0.15)	<.001	0.13 (0.11-0.15)	<.001	.73
Model 2[b]	0.10 (0.09-0.12)	<.001		0.10 (0.08-0.12)	<.001	0.11 (0.08-0.13)	<.001	.75
Model 3[c]	0.10 (0.09-0.12)	<.001		0.10 (0.08-0.12)	<.001	0.10 (0.08-0.13)	<.001	.76
Model 4[d]	0.09 (0.08-0.11)	<.001		0.09 (0.07-0.11)	<.001	0.10 (0.08-0.12)	<.001	.74
**ADL limitation**								
Model 1[a]	0.10 (0.08-0.12)	<.001		0.10 (0.08-0.12)	<.001	0.10 (0.08-0.13)	<.001	.66
Model 2[b]	0.08 (0.07-0.10)	<.001		0.08 (0.06-0.10)	<.001	0.09 (0.06-0.11)	<.001	.64
Model 3[c]	0.08 (0.07-0.10)	<.001		0.08 (0.06-0.10)	<.001	0.09 (0.06-0.11)	<.001	.61
Model 4[d]	0.08 (0.06-0.09)	<.001		0.07 (0.05-0.09)	<.001	0.08 (0.06-0.10)	<.001	.55
**IADL limitation**								
Model 1[a]	0.12 (0.11-0.14)	<.001		0.13 (0.10-0.15)	<.001	0.12 (0.10-0.14)	<.001	.66
Model 2[b]	0.09 (0.08-0.11)	<.001		0.10 (0.08-0.12)	<.001	0.09 (0.07-0.11)	<.001	.70
Model 3[c]	0.09 (0.08-0.11)	<.001		0.10 (0.07-0.12)	<.001	0.09 (0.07-0.11)	<.001	.66
Model 4[d]	0.09 (0.07,0.10)	<.001		0.09 (0.07-0.11)	<.001	0.09 (0.07-0.11)	<.001	.65

[a]: Model 1 was not adjusted for any covariates; [b]: Model 2 was adjusted for covariates including individual's age, residence, region, occupation, education, income, and health insurance; [c]: Model 3 was adjusted for covariates including individual's age, residence, region, occupation, education, income, health insurance, social activities, smoking, and drinking; [d]: Model 4 was adjusted for covariates including individual's age, residence, region, occupation, education, income, health insurance, social activities, smoking, drinking, self-rated health, and multimorbidity; [e]: In models for the total sample here, gender was additionally added to the models as an adjustment variable (Models 1-4). [f]: *P* for gender interaction was examined using the gender-by-functional limitation (or ADL/IADL limitation) interaction test.
Abbreviations: CI=confidence interval

© 2021 Wang J et al. *JAMA Network Open.*

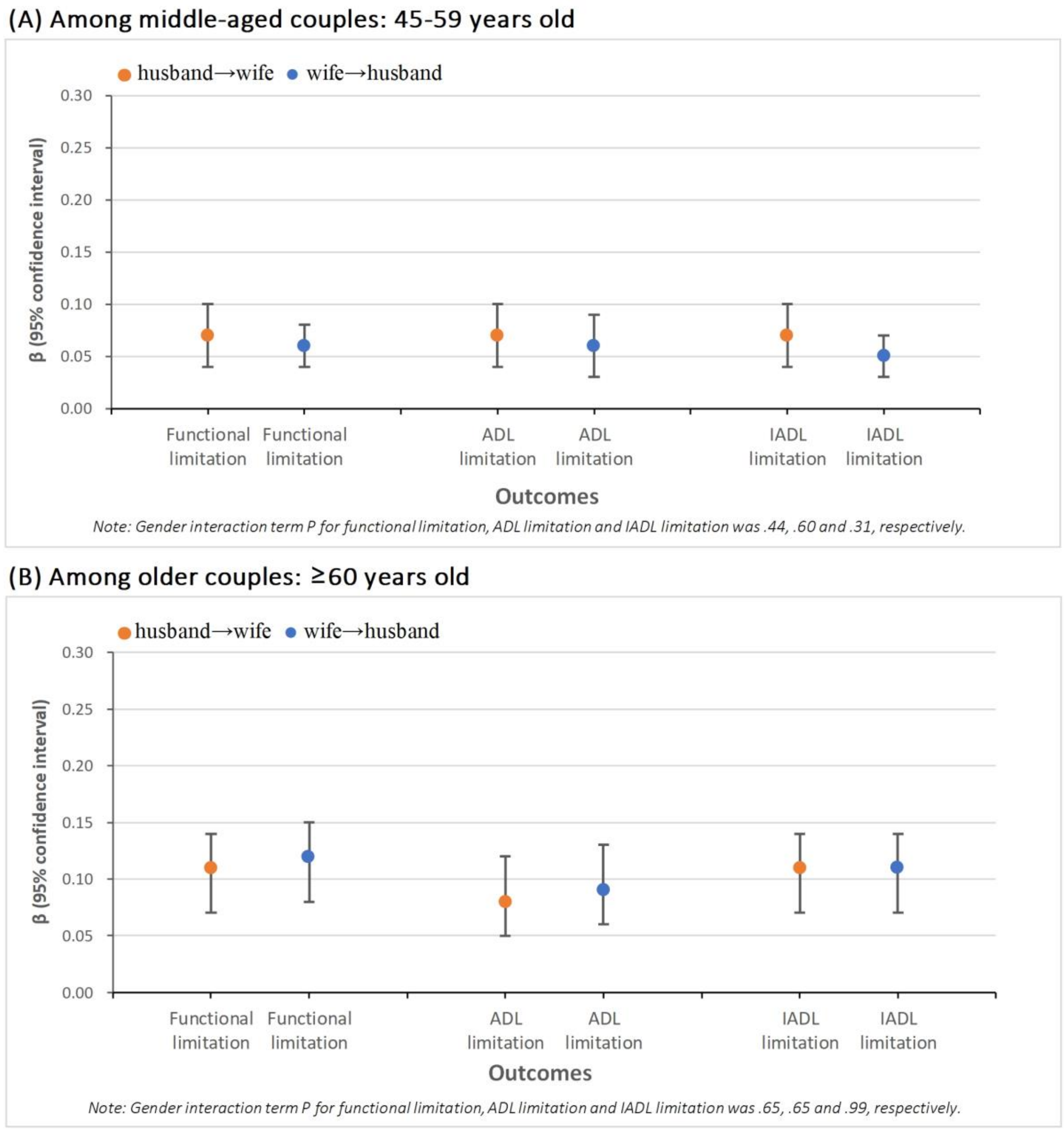

eFigure 1. Reciprocal Association in Functional Limitation (as Continuous Scores) by Gender Among Different Age Groups, 2011-2018

Note: All models were adjusted for individual's residence, region, occupation, education, income, health insurance, social activities, smoking, drinking, self-rated health and multimorbidity. Gender interaction term *P* was obtained using the gender-by-functional limitation (or ADL/IADL limitation) interaction test.

© 2021 Wang J et al. *JAMA Network Open.*
